# Inactivation of *Staphylococcus aureus* by antimicrobial photodynamic therapy using 1,9-Dimethyl-Methylene Blue: in vitro and in vivo studies

**DOI:** 10.1007/s10103-025-04362-6

**Published:** 2025-02-25

**Authors:** Anildo Alves de Brito Júnior, Pedro Jorge Louro Crugeira, Andressa Vollono Barbosa, Wellington Luis Reis Costa, Maria Cristina Teixeira Cangussu, Susana Carla Pires Sampaio de Oliveira, Amanda Inês Vieira de Mello, Antônio Luiz Barbosa Pinheiro, Juliana Monteiro Azevedo

**Affiliations:** 1 Center of Biophotonics, Faculty of Dentistry, Federal University of Bahia, 62, Araujo Pinho Ave, Canela, Salvador, BA CEP 40110-150 Brazil; 2 Centro de Investigação de Montanha (CIMO), Instituto Politécnico de Bragança, Campus de Santa Apolónia, Bragança, 5300-253 Portugal; 3 Laboratório Associado Para a Sustentabilidade e Tecnologia em Regiões de Montanha (SusTEC), Instituto Politécnico de Bragança, Campus de Santa Apolónia, Bragança, 5300-253 Portugal; 4 Dentistry and Health Postgraduate Program, School of Dentistry, Federal University of Bahia, Salvador, BA 40110-150 Brazil; 5 Department of Biology, Feira de Santana State University, Feira de Santana, BA 44036-900 Brazil; 6 Center of Biophotonics, Faculty of Dentistry, Federal University of Bahia, 62, Araujo Pinho Ave, Canela, Salvador, BA CEP 40110-150 Brazil; 7 Faculty of Dentistry, Federal University of Bahia, 62, Araujo Pinho Ave, Canela, Salvador, BA CEP 40110-150 Brazil; 8 Feira de Santana State University, Av. Transnordestina, s/n, Feira de Santana, Novo Horizonte, BA CEP 40110-150 Brazil; 9 Center Research Mountain (CIMO), Institute of Polytechnic Institute of Bragança, Santa Apolónia Campus, Bragança, 5300-253 Portugal

**Keywords:** *Staphylococcus aureus*, Photochemotherapy, Photosensitizing drugs, LED

## Abstract

The efficiency of antibiotics in terms of their bacterial inhibition is well known. However, studies show that its overuse, underuse, and misuse induce antimicrobial resistance, promoting the need to work with alternative methods. In this sense, antimicrobial photodynamic therapy (aPDT) is a promising selective method demonstrating excellent response. This study aimed to evaluate the antimicrobial action promoted in *Staphylococcus aureus* using 1,9 dimethyl methylene blue dye (DMMB) combined with red LED (λ 630 ± 20 nm, CW, 125 mW, 12 J/ cm², 192 s) in planktonic culture and rats skin wounds contaminated with staphylococcal bacteria. The experimental in vitro and in vivo groups were Control, LED, DMMB, and LED + DMMB; after aPDT, the triplicate samples for each dilution were incubated for 24 h, and the number of bacteria was determined by counting the colony-forming units, and the logarithm (CFU/mL log). Based on in vitro data obtained, the LED + DMMB group, when compared to the Control, showed a reduction in microbial load of 99.943% (*p* < 0.0001), with decimal reduction (RD = 3). Whereas in vivo results, the same comparing groups demonstrated a reduction in microbial load, reaching 99.994% (RD = 4). In this research, the aPDT was a unique treatment, and it is possible to repeat it to obtain higher microbial reduction, providing an alternative therapeutic that can be clinically validated to combat infections caused by *S. aureus*.

## Introduction

The global prevalence of chronic wounds has a significant impact on the health care system due to economic burden, but also clinically and socially [1]. Wound chronicity is associated with colonizing pathogenic bacteria at the wound site. It causes a delay in the healing process [2]. S. aureus can be present in human skin and mucous membranes as a member of the normal microbiota, but in some conditions, it acts as a pathogen, and due to its easy dissemination it can cause everything from abscesses to sepsis [3–5]. Although the efficiency of antibiotics in terms of their bacterial inhibition is well known, their overuse, underuse, and misuse have increased the antimicrobial resistance process [6]. About 90% of S. aureus strains resist various antibiotics, reducing antibiotic effectiveness [7], complicating the treatment, and a working vaccine is unavailable [3].

Therefore, alternative strategies to control S. aureus infection have gradually become the focus [8–9], such as antimicrobial photodynamic therapy (aPDT) [10, 11]. Different from antibiotics, which are against specific targets, the aPDT is an approach with multiple targets. Its mechanism of action can simultaneously damage several vital molecules and structures, such as proteins, nucleic acids, and membranes. Several pathways in intracellular metabolism are affected by aPDT, so it is unlikely that bacteria can develop resistance to this therapy [12].

The aPDT effects occur due to light, photosensitizing molecules (PS), and the presence of oxygen [13]. These PS will absorb visible light energy of a specific wavelength and subsequently produce reactive oxygen species (ROS), which could initiate different cell death pathways: apoptosis, necrosis, and autophagy in target tissues [14]. ROS synthesis can be based on electron transfer to produce superoxide or through energy transfer that will produce highly reactive singlet oxygen [15]. The ROS promotes photo-oxidative stress on organic molecules in the nanometer vicinity of the PS, such as lipids and proteins, which comprise the bacterial envelope’s basilar structure. Then, it causes bacterial death [8–16].

Among the available PSs, the phenothiazinium chromophores employed in aPDT are methylene blue (MB), toluidine blue (TB), and phenothiazinium derivatives such as new methylene blue (NMB) and dimethyl-methylene blue (DMMB) [17–20]. Nowadays, ideal PS should combine some properties, among which are to be inactive in the absence of light (no toxicity or mutagenicity) and to generate a 1O2 high quantum yield [21, 22]. DMMB stands out as more resistant to reduction to its inactive leuco form, and by being able to produce higher levels of singlet‑oxygen when compared to others [23]. The efficiency of DMMB is increased considerably because of two extra methylene groups [24]. However the exact site of their generation is more important than the amount of oxidant species [25, 26]. In this way, DMMB inserts deeper into the membrane bilayer, then consequently, photo irradiation of DMMB causes a substantial release of carboxyfluorescein from small unilamellar vesicles, indicating membrane permeabilization; therefore, photo damages its targets more precisely than the others phenothiazinium PSs [23].

DMMB’s higher phototoxicity and sites of intracellular action might suggest that DMMB could be a more efficient PS when compared to others [26] Although a few studies showed the action of DMMB against planktonic bacteria S. aureus [27–28], no in vivo studies were found in actual literature, which encouraged this present research. In light of the potential DMMB applicability for staphylococcal infections, the objective is to evaluate the effects of the aPDT against S. aureus, both in vitro and in vivo experimental models by DMMB irradiated with red LED.

## Materials and methods

### Photosensitizer

Experimental in vitro and in vivo procedures used 1,9-Dimethyl-Methylene Blue zinc chloride double salt powder with 80% dye content as photosensitizer, also known as Taylor’s Blue; it was purchased from Sigma (Sigma-Aldrich, St. Louis, MO, USA), with an absorbance at λ_máx_ 649 nm. The average inhibitory concentration (IC_50_) of the DMMB photosensitizer in the *S. aureus* ATCC 25,923 strain was determined according to the results of previous studies by our group (340.5 ng/mL) [27–28].

### Light source

The light source used in this study was an LED device (Fisioled^®^, MMOptics, São Carlos, SP, Brazil, λ630 ± 20 nm, 125 mW, CW, 192 s, 2.0 cm^2^, 12 J/cm^2^). The equipment was calibrated correctly before the experiment using a power meter (Thorlabs PM30, Newton, NJ, USA).

### Bacterial strain

The tests used the *S. aureus* (ATCC 25923) strain acquired from Labchecap (Lab and Image, Salvador, Brazil). For experimental purposes, *S. aureus* was removed from the ultra-freezer (Thermo Electron Corporation, Bartlesville, OK 74003, USA) to restore metabolic activity and suitability for the carbon source. First, the bacteria grew aerobically in brain heart infusion broth (BHI^®^, Sigma-Aldrich, Germany) in a bacteriological oven (TE 391/1^®^ TECNAL, Brazil) for 12 h at 37 °C. The culture was diluted in tryptic soy broth (TSB, Merck^®^, Darmstadt, Hessen, Germany) and incubated overnight. To confirm the culture activation, it was used Baird-Parker agar (DifcoTM Baird-Parker Agar Base ref. 0768 + DifcoTM Egg Yolk Enrichment 50% ref. 3347), which is a selective medium used for the isolation and differentiation of coagulase-positive staphylococci and it was realized Gram stain (shape, size, and arrangement).

The standardization of the bacterial inoculum used in vitro and in vivo procedures was adjusted in a microplate spectrophotometer (SpectraMax^®^190 Molecular Device, California - USA) to obtain an optical density (OD) corresponding to 0.5 of the McFarland standards, with turbidity equivalent to an approximate concentration of bacteria of 1.5 × 10^8^ colony-forming units (CFU/mL).

### In vitro photodynamic inactivation of *Staphylococcus aureus*

All aPDT procedures were performed in a laminar flow hood with minimal exposure to light. The 24-well plates (Falcon^®^, BD Lab., Franklin Lakes, New Jersey, USA) were used for each experimental group, containing the bacterial inoculum standardized (approximate concentration of bacteria of 1.5 × 10^8^ CFU/mL) and phosphate-buffered saline (PBS, Gibco^®^, USA). Then, the samples were divided into Control, LED, DMMB, and LED + DMMB groups (Table 1).

In the DMMB and LED + DMMB groups, 300 ng/mL of the DMMB photosensitizer was used, and the pre-irradiation time was five minutes in the dark and at room temperature. The LED and LED + DMMB groups were irradiated according to the LED parameters.

After irradiation, the samples were diluted in series 10^− 1^ to 10^− 9^ (1:10 at a time). 100 µL aliquots of each dilution (10^− 1^ to 10^− 9^) for each group (Control, LED, DMMB, and LED + DMMB) were seeded in triplicate on tryptic soy agar (TSA, Merck^®^, Darmstadt, Hessen, Germany) in Petri dishes. Then, the Petri dishes were incubated in a bacteriological oven (TE-391/1^®^ TECNAL, Brazil) at 37ºC for 24 h to count the colony-forming units and the log of CFU/mL (Log _10_ CFU/mL) calculated.

### In vivo photodynamic inactivation of *Staphylococcus aureus*

The Ethics Commission for the Use of Animals (CEUA/ICS/UFBA) approved this study (7290250722). The Central Animal Facility of the Faculty of Veterinary Medicine of UFBA provided twelve male *Wistar* rats with an average age of three to four months and weight of 250 to 300 g. All procedures were conducted in the Animal Experimentation Laboratory of the Faculty of Dentistry of the UFBA. The animals were distributed into four experimental groups, with three animals in each experimental group according to Table 1.

**Table 1:** Experimental in vitro and in vivo groups

Groups	In vitro Treatments	In vivo Treatments
Control	No treatment. Staphylococcal bacteria (1.5 × 10^8^ CFU/mL)	No treatment. Surgical skin wounds contaminated with Staphylococcal bacteria (1.5 × 10^8^ CFU/mL). *n* = 3 animals
LED	Staphylococcal bacteria irradiated by LED (λ 630 ± 20 nm, CW, 125 mW, 12 J/cm ², 192 s).	Surgical skin wounds contaminated with Staphylococcal bacteria irradiated by LED (λ 630 ± 20 nm, CW, 125 mW, 12 J/cm ², 192 s). *n* = 3 animals
DMMB	Staphylococcal bacteria treated by DMMB (300 ng/mL).	Surgical skin wounds contaminated with Staphylococcal bacteria treated by DMMB (300 ng/mL). *n* = 3 animals
LED + DMMB	Staphylococcal bacteria were treated by DMMB (300 ng/mL) and irradiated by LED (λ 630 ± 20 nm, CW, 125 mW, 12 J/cm², 192 s).	Surgical skin wounds contaminated with Staphylococcal bacteria treated by DMMB (300 ng/mL) and irradiated by LED (λ 630 ± 20 nm, CW, 125 mW, 12 J/cm², 192 s). *n* = 3 animals

After weighing, trichotomy, local antisepsis with a 2% chlorhexidine solution (Maquira, DentalSpeed, Brazil), the animals were anesthetized with a 5 mg/kg Xylazine solution (10% Dopase^®^, São Paulo, Brazil) and 80–100 mg/kg of ketamine (Duralay^®^ Reliance, Dental MFG co-Worth, IL, USA) intramuscularly, subsequently undergoing the surgical procedure to create the wound.

One circular symmetrical marking of 10 mm in diameter was made on the back of each rat in the midline with a punch (Starfer^®^, Amazon, Brazil). The surgical incision was made with a scalpel nº15, following the demarcation, and the size of the wound was confirmed with a caliper (Mitutoyo, São Paulo, Brazil) [29, 30]. The wound reached the skin, cutaneous muscle, and subcutaneous fat, maintaining a wound depth of 1 mm in all animals [31]. A single calibrated operator performed the entire surgical procedure.

The skin wounds were inoculated with 200 µL aliquots standardized of *S. aureus* (approximate concentration of bacteria of 1.5 × 10^8^ CFU/mL) [29]. 24 h after contamination of the wounds by *S. aureus*, secretions were collected from the wounds of all animals in all groups to confirm the infection. The swabs were placed in sterile tubes with 2 mL of Phosphate-buffered saline (PBS, Gibco^®^, USA) and identified. After diluted in series 10^− 1^ to 10^− 9^ (1:10 at a time) in the same sterile diluent, 30 µL aliquots of each dilution (10^− 1^ to 10^− 9^) for each animal were seeded in triplicates by dissemination with a Drigalsky loop in a selective medium for *S. aureus* (Baird-Parker Agar Base^®^ + 50% Egg Yolk Enrichment^®^, Difco) in tripartite (two divisions) Petri dishes. Then, the Petri dishes were inverted and incubated in a bacteriological oven (TE-391/1^®^ TECNAL, Brazil) at 37ºC for 24 h to count the colony-forming units.

48 h after wound contamination with *S. aureus*, after confirmation of the infection of each animal, the proposed treatment was conducted in all groups. The animals were anesthetized in the DMMB and LED + DMMB groups it was used 200 µL aliquots of DMMB (300 ng/mL) were placed directly onto the contaminated skin wounds, and the pre-irradiation time was five minutes. The LED and LED + DMMB groups were irradiated according to the LED parameters (λ 630 ± 20 nm, CW, 125 mW, 12 J/cm ², 192 s), the LED tip spot covered the entire skin wound, and the irradiation was done at an angle of 90º.

Immediately, the secretions were collected from the animals’ wounds, and the microbiological analysis followed the methodology described to count the colony-forming units. All in vivo experiments were performed in triplicate, then for in vivo procedures, 12 animals resulted 36 samples. Three samples were collected from each animal, each sample was serially diluted and plated in triplicate, and this means that for each experimental group an average of the nine replicates was obtained.

### Statistical analysis

Statistical analysis was performed using the GraphPad^®^ Prism software (San Diego-CA, USA). Values ​​expressed as Log10 means were analyzed using the one-way ANOVA test and Tukey’s multiple comparisons. For all analyses, p values ​​<0.05 were considered statistically significant.

## Results

The *S. aureus* strain was confirmed through the Grey-black colonies and a halo on Baird-Parker agar; it indicates coagulase-positive staphylococci. Gram stain procedure showed Gram-positive bacteria.

**Fig. 1:**
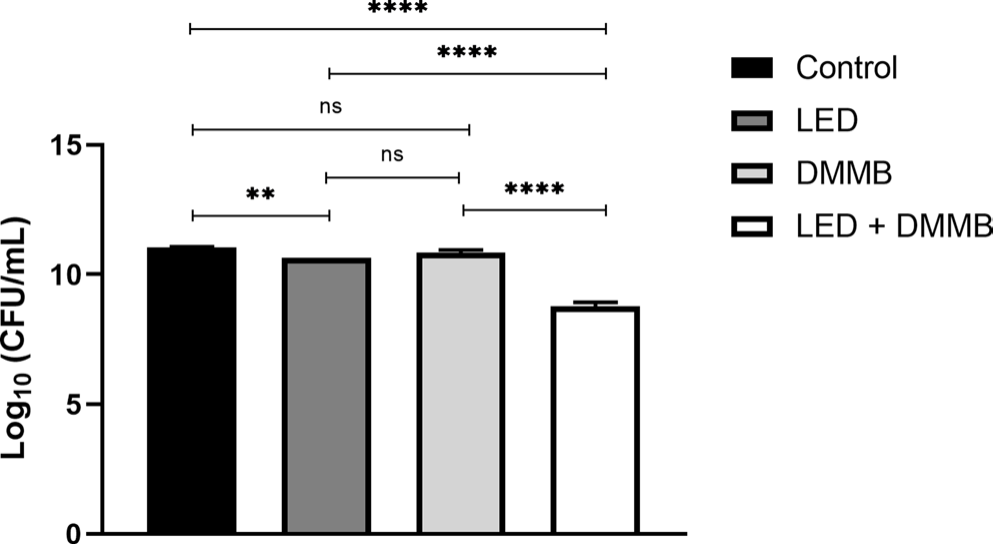
Graphic representation of ANOVA and Tukey statistical analysis of in vitro study between Control, DMMB, LED, and aPDT (LED + DMMB) groups. ns (no significant), *** *p* < 0.001 **** *p* < 0.0001. *n* = 12

**Fig. 2:**
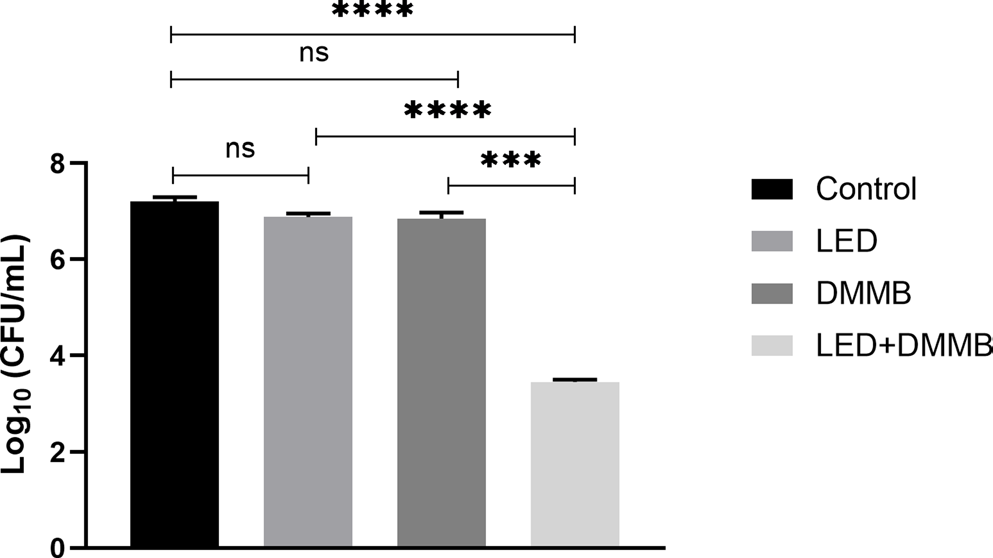
Graphic representation of ANOVA and Tukey statistical analysis of in vivo study between Control, DMMB, LED, and aPDT (LED + DMMB) groups. ns (no significant), *** *p* < 0.001 **** *p* < 0.0001. *n* = 36

The mean values ​​of the colony-forming unit count of in vitro and in vivo photodynamic inactivation of *S. aureus* were expressed as the logarithm (CFU/mL log). The data obtained in all the in vitro experimental conditions studied can be seen in Fig. 1, and in vivo data in Fig. 2.

Based on in vitro data obtained, photodynamic therapy for the inactivation of *S. aureus* (LED + DMMB) showed a significant reduction (*p* < 0.0001) in microbial load, reaching 99.943% (DR = 3) when compared to the Control group. Whereas in vivo results comparing the same groups demonstrated decimal reduction (DR = 4) reaching 99.994%, a significant decrease (*p* < 0.0001) in microbial load in the LED + DMMB group.

In the in vitro LED group, a significant reduction (*p* < 0.01) in the microbial load of *S. aureus* reaching 61.50% was seen when compared to the Control group, while in vivo, data obtained was not statistically significant because the percentage reduction reached only 53.53 in microbial load in the LED group compared to the Control group.

The action of the photosensitizer used alone in the in vitro and in vivo DMMB group demonstrated a non-significant reduction in the population of *S. aureus* compared to the respective Control groups.

## Discussion

Disruption of the skin causes alterations in the structural integrity and functional continuity; when this barrier is lost, the sub-epidermal tissues are exposed to the external environment, and bacterial colonization may occur [32]. The infection reduces the possibility of the wound healing properly; the healing process fails, and the wounds can be considered chronic after six weeks. Recent studies related *S. aureus* as a cause of chronic wound infections [32–33].

The effective treatment of staphylococcal infections remains a significant public health challenge, including clinical and social impacts and economic burdens. It occurs due to the resistance mechanisms of bacteria against traditional antimicrobial agents because it requires additional therapeutic procedures that increase the cost of treatment [1, 7]. Therefore, recent research highlights several preventive and therapeutic innovative strategies to combat *S. aureus* infections, including the aPDT [10, 11].

In this sense, the present investigation demonstrated the effects of aPDT against *S. aureus* by DMMB (300 ng/mL) irradiated with red LED (λ630 ± 20 nm, CW, 125 mW, 2.0 cm^2^,12 J/cm², 192s). The principal result showed a 99.994% reduction in surgical skin wounds contaminated with staphylococcal bacteria. Although a more significant challenge is expected in the in vivo experimental model, the complexity of the biological system and the action of the host’s immune system must be considered [34].

The DMMB was inserted in the aPDT protocol previously studied and established by our research group both in vitro [27, 28, 35–38] and in vivo, including a recent investigation about the efficiency of aPDT employing DMMB as a photosensitizing agent combined with red LED irradiation (λ640 ± 5 nm) against oral biofilm of patients undertaking orthodontic treatment [39].

In the present study, both in vitro and in vivo experiments, comparing DMMB groups with respective Control groups, the action of the PS used alone demonstrated no significant reduction in the population of *S. aureus*. This result is expected due to the use of the PS in a concentration (300 ng/mL) below the IC_50_ (340.5 ng/mL), corroborating with previous studies [27, 28]. An in vitro study demonstrated excellent antimicrobial properties of DMMB against *S. aureus* using higher than 1 µg/ml concentration [40]. The FS remains inactive without light; ROS should not be generated without irradiation, according to ideal FS properties, which are non-toxic without light [21, 22]. Therefore, it is remarkable in the present research that DMMB in nano concentration showed no toxicity to the microorganism used alone. It only had a cytotoxic effect when irradiated with the LED light source. This corresponds to the characteristics of the ideal FS: it has the lowest possible concentration, is not toxic in the dark, and its antimicrobial effect only occurs in the presence of light.

In planktonic cultures, comparing the LED group (light source alone) with the Control group, there was a significant decrease (*p* < 0.01) (61.50%) in the microbial load of *S. aureus* irradiated. It should be noted that there is a discussion in the literature about the isolated action of light because the stimulation or inhibition of the microbial load depends on the species of bacteria and wavelength of irradiation, as well as other parameters related to the light source. It demonstrated a stimulatory effect of laser irradiation on *S. aureus* by infrared light (λ904 nm, 27 W, 0.03 J/cm^2^, 6000 Hz) [41]. An inhibitory effect could be promoted by photodynamic damage justified by electronic excitation with energy transfer to the O_2_ of the medium since the cytochrome acts as an FS [38].

On the other hand, in vivo data obtained when the LED group was compared to the Control group demonstrated a non-statistically significant result, the percentual reduction reaching only 53.53 in microbial load. It is possible that LED irradiation in vitro directly affects only planktonic cultures, but in vivo, it also alters intrinsic cellular activity via absorption by chromophores in the skin. Photobiomodulation has a positive effect on the healing of infected wounds, red and infrared lights, as well as restores immunosuppression and improves immunity [42, 43].

The bactericidal effect of the aPDT against S. aureus was observed in the in vitro LED + DMMB group, with a reduction of the microbial load reaching 99.943% (*p* < 0.0001) compared to the Control group. Our results corroborate a previous study [28] using another light source (polarized light), which demonstrated a 99.97% reduction in the microbial load of *S. aureus* by DMMB-aPDT (300 ng/mL) associated with polarized light (λ400–2000 nm, 5 J/cm^2^). In addition, it agrees with another study [30] that demonstrated a 99.96% reduction in the microbial load of *S. aureus* by DMMB-aPDT (300 ng/mL) associated with red LED (12 J/cm^2^). The present study is also in agreement with previous studies that used phenothiazine compounds (methylene blue and toluidine blue) as a photosensitizer at a concentration of 12.5 µg/mL associated with red light (red-orange λ632 ± 20 nm, 145 ± 5 mW, 12 J/cm^2^) against *S. aureus* population reaching reduction in the microbial load [35–36].

Comparing LED + DMMB to the Control group, the in vivo results demonstrated a 99.994% reduction in the microbial load of *S. aureus*. This enhanced result can be attributed to the ability of aPDT to modulate the host response and improve immunity per significant reduction in expression of pro-inflammatory cytokines TNF-α, IL-6, IL-8 [43, 44]. Evidence in the literature suggests that aPDT leads to an increase in the immunomodulatory activity of the tissue by decreasing T lymphocyte stimulus and influencing immune stimulatory properties of antigen-presenting cells [45].

The efficiency of aPDT does not depend only on the amount of ROS generated by the reaction; the specific place of its generation is too important [18, 23]. DMMB has a positive charge and greater lipophilicity (compared to MB); these characteristics increase affinity to S. aureus, a Gram-positive coccus with teichoic and lipoteichoic acids in its cell wall [39, 42]. Excellent results about the DMMB-aPDT against other gram-positive bacteria were also observed in the planktonic culture of *Enterococcus faecalis*, reducing 99.999998% of the microbial load using 3.32 ng/mL of DMMB associated with LED light (λ632 ± 20 nm, 18 J/cm^2^) [37].

The therapeutic proposal of the present study demonstrated promising results, with potential for application in clinical studies and health services. The light source employed presents the advantages of effectiveness and low cost, and the FS was used in nano concentration. Regarding treating chronic wounds mediated by *S. aureus*, in which conventional therapies have limitations, the use of DMMB-aPDT can be used as a therapeutic alternative or adjuvant. Remarkably, the protocol used was a single application; it is possible to repeat it, achieving even better results in reducing the microbial load. As a limitation of this study, it is highlighted that it is a treatment with a topic effect. More studies are needed to evaluate the aPDT response to other challenges, such as systemic conditions in the host and mineralized tissues. The sample population can be expanded in future researches to further test, then clinical validation will equip the medical field with alternative treatment methods against staphylococcal infections.

## Conclusion

The findings of the present research demonstrated the effectiveness of aPDT against *S. aureus* by DMMB phenothiazine dye irradiated with LED light (λ630 ± 20 nm, CW, 125 mW, 2.0 cm^2^, 12 J/cm², 192s) with a result of 99.943% reduction in microbial load in planktonic culture, and 99.994% reduction in surgical skin wounds contaminated with staphylococcal bacteria. They provided an alternative therapeutic option that can be better investigated and clinically validated to combat infections caused by bacterial strains of *S. aureus*.
